# Advances in Understanding Inflammation and Tissue Damage: Markers of Persistent Sequelae in COVID-19 Patients

**DOI:** 10.3390/jcm14051475

**Published:** 2025-02-22

**Authors:** Raul Patrascu, Cristina Stefania Dumitru

**Affiliations:** 1 Department of Functional Sciences, “Victor Babes” University of Medicine and Pharmacy, 300041 Timisoara, Romania; 2 Department of Microscopic Morphology/Histology, “Victor Babes” University of Medicine and Pharmacy, 300041 Timisoara, Romania; cristina-stefania.dumitru@umft.ro

**Keywords:** post-COVID-19 conditions, biomarkers, inflammation, tissue damage, longitudinal studies, long COVID

## Abstract

This review explores the crucial role of established and emerging biomarkers in the diagnosis, management, and understanding of post-COVID-19 conditions. With COVID-19 affecting multiple organ systems, biomarkers have been instrumental in identifying ongoing inflammation and tissue damage, facilitating early diagnosis and prognostication. Specifically, markers like C-reactive protein (CRP), interleukin-6 (IL-6), and novel entities such as soluble urokinase plasminogen activator receptor (suPAR) and neutrophil extracellular traps (NETs) provide insights into the pathophysiological mechanisms and predict long-term outcomes. This review highlights the integration of these biomarkers into clinical workflows and their implications for personalized medicine, emphasizing their potential in guiding therapeutic interventions and monitoring recovery. Future directions suggest a focus on longitudinal studies to explore biomarker trajectories and their interaction with therapeutic outcomes, aiming to enhance the management of post-COVID-19 conditions and refine public health strategies.

## 1. Introduction

Since the emergence of Severe Acute Respiratory Syndrome Coronavirus 2 (SARS-CoV-2) in late 2019, the resulting COVID-19 pandemic has inflicted unparalleled public health crises and economic disruptions worldwide. As of mid-2023, the virus has affected over 600 million individuals globally, culminating in more than 6.5 million deaths. The spread of this virus has not only placed unprecedented stress on healthcare systems but also exposed significant vulnerabilities in global health governance and emergency preparedness. Recent studies highlight these challenges, including the critical role of novel biomarkers in identifying severe cases and predicting outcomes [1], and the potential molecular pathways involved in severe complications such as pneumomediastinum, which could inform future therapeutic strategies [2]. These insights underscore the need for robust health policies and advanced research to better prepare for and mitigate the impacts of current and future pandemics.

While the acute respiratory symptoms of COVID-19 are well documented, increasing attention is being directed towards the post-acute sequelae of SARS-CoV-2 infection (PASC), commonly known as long COVID [3]. This condition encompasses a range of symptoms and medical complications that persist for weeks to months after the initial recovery from the acute phase of the illness. PASC affects a significant portion of COVID-19 survivors, with symptoms including, but not limited to, chronic fatigue, respiratory difficulties, cognitive impairments, and cardiovascular abnormalities, which underscore the virus’s capability to inflict systemic and multi-organ damage [4].

The ongoing research into PASC highlights the critical role of immunological markers in understanding the extended impact of the virus. Identifying and characterizing these markers is essential for elucidating the pathophysiological mechanisms underlying persistent symptoms and guiding long-term patient management strategies [5]. Moreover, these markers can provide valuable insights into the inflammatory processes and tissue damage that contribute to the severity and longevity of post-COVID-19 conditions, informing both therapeutic approaches and preventive measures in clinical practice [6].

The identification of specific biomarkers related to inflammation and tissue damage is crucial for the clinical management of PASC [7]. These biomarkers not only provide a quantifiable measure of biological processes but also assist in the stratification of risk among COVID-19 survivors, enabling personalized therapeutic interventions. By leveraging biomarker data, clinicians can predict potential complications, monitor disease progression, and adjust treatments accordingly, thus optimizing patient outcomes [8].

Recent advancements in biomarker research have led to the discovery of several novel markers that offer deeper insights into the inflammatory and pathophysiological processes associated with COVID-19. These include cytokines, chemokines, and other cellular markers that shed light on the underlying mechanisms driving long-term symptoms and tissue damage in PASC. The ongoing development of high-throughput technologies and bioinformatics tools further enhances our ability to identify and utilize these markers effectively in clinical settings [9,10].

This review aims to synthesize current knowledge on both common and novel biomarkers of inflammation and tissue damage, particularly in the context of PASC. By providing a comprehensive overview of these markers, their clinical relevance, and the potential they hold for improving patient care, this paper contributes to a better understanding of PASC and supports the development of targeted strategies for its management and prevention [11]. As new therapeutic options and diagnostic tools emerge, staying abreast of these biomarkers will be essential for all healthcare providers managing COVID-19 patients [12].

## 2. COVID-19 and the Immune Response

### 2.1. Initial Immune Defense Against SARS-CoV-2

The immune response to SARS-CoV-2 begins with the virus’s entry into the host cells via the ACE2 receptor, which is predominantly found in the respiratory tract [13]. Upon infection, the innate immune system responds swiftly, primarily through the activation of macrophages and dendritic cells that recognize viral components. These immune cells secrete type I interferons and pro-inflammatory cytokines, initiating an antiviral state in neighboring cells and recruiting additional immune mediators to the site of infection. This early response is crucial for containing the virus and preventing its spread, highlighting the body’s initial defense mechanisms against SARS-CoV-2 [14].

As the immune battle progresses, the adaptive immune system comes into play, characterized by the activation of T cells and B cells. T cells, once activated, perform a variety of functions; CD8+ T cells directly kill virus-infected cells, whereas CD4+ T cells assist in B cell maturation and antibody production. Concurrently, B cells differentiate into plasma cells, producing virus-specific antibodies that neutralize the virus and prevent further cell entry. The coordination between innate and adaptive immunity is critical for resolving the infection and developing long-term immunity, which is evidenced by the production of memory cells that provide lasting protection against the virus [15,16].

### 2.2. Dysregulation and Progression to Severe Disease

In some cases, the immune response to SARS-CoV-2 becomes dysregulated, leading to an excessive inflammatory response known as a cytokine storm [17]. This hyperinflammatory state is characterized by the overproduction of pro-inflammatory cytokines such as IL-6, TNF-α, and IL-1β, which can cause widespread tissue damage and contribute to the severe manifestations of COVID-19, such as acute respiratory distress syndrome (ARDS). The factors leading to this dysregulation are multifaceted, involving genetic predispositions, underlying health conditions, and possible viral load. Understanding the transition from a protective immune response to a pathological one is essential for identifying therapeutic targets to prevent severe disease [18].

### 2.3. Chronic Inflammation and Its Consequences

Chronic inflammation post-COVID-19 infection is a pivotal factor contributing to prolonged and recurrent symptoms, a hallmark of post-acute sequelae [19]. This persistent inflammatory state is characterized by the continued activation of immune cells and the release of inflammatory mediators long after the acute infection has been resolved. Studies indicate that such chronic inflammation can lead to scarring and long-term damage in organs like the lungs, heart, and brain, which correlates with symptoms like breathlessness, fatigue, and cognitive dysfunction seen in long COVID patients [20].

Recent research has focused on identifying specific immunological markers that can signal ongoing inflammation and predict long-term health outcomes. Markers such as elevated levels of C-reactive protein (CRP), D-dimer, and sustained elevation of cytokines like IL-6 have been associated with poorer prognoses and prolonged recovery times [21]. These markers are not only instrumental in diagnosing and monitoring post-COVID-19 conditions but also serve as potential targets for anti-inflammatory therapies aimed at mitigating long-term effects [22].

The current advancements in immunological research are providing new insights into the mechanisms of PASC and opening pathways for targeted interventions. Studies employing advanced technologies such as single-cell RNA sequencing and proteomics are elucidating the cellular and molecular landscapes during and post-infection, offering a more detailed understanding of the prolonged immune response. Such insights are crucial for developing targeted therapies that can modulate the immune response, prevent severe sequelae, and improve the quality of life for those affected by long COVID [23,24].

## 3. Markers of Inflammation in Post-Acute COVID-19

### 3.1. Established Inflammatory Markers in Post-COVID-19

In the landscape of post-acute COVID-19, certain established inflammatory markers have been instrumental in monitoring ongoing inflammation and predicting long-term outcomes. C-reactive protein (CRP), a marker commonly associated with acute inflammation, continues to be elevated in many patients experiencing post-COVID-19 syndromes, indicating persistent inflammatory activity [25]. Similarly, elevated levels of serum ferritin and erythrocyte sedimentation rate (ESR) have been correlated with prolonged recovery phases and increased severity of post-acute symptoms. Understanding the role of these markers in the clinical trajectory of COVID-19 survivors helps in guiding management strategies and therapeutic interventions [26].

### 3.2. Novel Biomarkers of Inflammation

Emerging research has identified novel biomarkers that offer deeper insights into the pathophysiological mechanisms underlying post-COVID-19 conditions. Among these, already well-known cytokines such as interleukin-6 (IL-6) and tumor necrosis factor-alpha (TNF-α) have been noted for their roles in chronic inflammation and tissue damage [27]. Additionally, soluble urokinase plasminogen activator receptor (suPAR) has gained attention for its predictive value in determining disease progression and outcomes in COVID-19 patients. These biomarkers not only enhance our understanding of disease processes but also open new avenues for targeted therapies aimed at modulating inflammatory responses in affected individuals [28].

Chemokines play a critical role in orchestrating the immune response during and after acute viral infections. In the context of COVID-19, chemokines such as CCL2 (MCP-1) and CXCL10 (IP-10) have been identified as key players in mediating immune cell migration and activation, which are crucial in the development and persistence of inflammation [29]. Elevated levels of these chemokines have been associated with severe disease during the acute phase and are believed to contribute to the chronic inflammatory state seen in PASC. Tracking these chemokines can provide valuable insights into the inflammatory status and guide therapeutic interventions to mitigate long-term effects [30].

### 3.3. Systemic Inflammatory Markers and Organ-Specific Damage

The correlation between systemic inflammatory markers and specific organ damage is of particular interest in post-COVID-19 management. Elevated levels of IL-10 and TGF-β, typically involved in anti-inflammatory responses, have paradoxically been linked with worse outcomes in COVID-19, suggesting a complex role in fibrosis and organ healing processes [31]. Research into how these cytokines influence recovery, particularly in the lungs and cardiovascular system, can help identify patients at risk of developing chronic conditions and enable earlier intervention strategies [32].

Recent studies have highlighted the role of neutrophil extracellular traps (NETs) in the pathology of severe COVID-19. NETs, which are web-like structures of DNA released by neutrophils during infection, contribute to inflammation and thrombosis, which are common complications in severe cases [33]. Biomarkers associated with NETosis, such as citrullinated histone H3 (Cit-H3), could serve as novel targets for therapeutic intervention to reduce the risk of thrombosis and improve patient outcomes in post-acute COVID-19 scenarios [34].

### 3.4. Advances in Biomarker Detection Technologies

The detection of these biomarkers has been enhanced by advances in technology, including high-sensitivity assays and multiplex platforms that allow for the simultaneous measurement of multiple cytokines and chemokines [35]. These technologies facilitate a more comprehensive understanding of the inflammatory landscape in COVID-19 patients and enable the development of tailored treatment plans based on specific biomarker profiles. The integration of machine learning models with biomarker data is also emerging as a powerful approach to predicting disease severity and outcomes in COVID-19 patients, paving the way for precision medicine in infectious diseases [36].

### 3.5. Clinical Implications and Future Directions

Understanding the full spectrum of inflammatory markers in post-acute COVID-19 patients is critical for the development of next-generation therapeutics and for informing long-term care strategies [37]. As research progresses, these markers will likely redefine clinical pathways and protocols for managing long COVID, emphasizing a shift from generalized to personalized medical approaches. Ongoing studies will need to focus on longitudinal data to better understand the trajectory of these markers and their relationship with clinical recovery in diverse populations [38,39].

## 4. Tissue Damage and Long-Term Effects

The long-term consequences of COVID-19 have become a growing concern, particularly as persistent symptoms continue to affect patients months after acute infection. These complications, collectively referred to as long COVID, are driven in part by immune dysregulation, unresolved inflammation, and direct tissue damage. This section explores specific biomarkers that have been associated with long-term organ dysfunction across major physiological systems, including neurological, pulmonary, fatigue-related, and gastrointestinal complications.

### 4.1. Neurological Sequelae of Long COVID

Neurocognitive symptoms such as brain fog, memory impairment, and persistent headaches are common in long COVID patients. Biomarkers such as neurofilament light chain (NfL) and glial fibrillary acidic protein (GFAP) have been implicated in post-viral neuroinflammation and neuronal damage. Elevated levels of NfL, a marker of axonal injury, have been found in patients experiencing persistent neurological symptoms, suggesting ongoing neurodegeneration or blood–brain barrier dysfunction [40,41]. GFAP, a marker of astrocytic injury, has been associated with white matter changes in long COVID patients, reinforcing the role of neuroinflammation in persistent symptoms [42].

### 4.2. Pulmonary Sequelae and Fibrosis

Post-COVID-19 lung disease is characterized by persistent respiratory symptoms, reduced lung function, and, in severe cases, pulmonary fibrosis. Biomarkers such as Krebs von den Lungen-6 (KL-6) and surfactant protein D (SP-D) are indicative of alveolar epithelial damage and fibrotic remodeling. Elevated KL-6 levels have been observed in patients with prolonged respiratory impairment, reflecting ongoing fibrotic processes similar to those seen in interstitial lung diseases [43,44]. SP-D, which is secreted by alveolar epithelial cells, has been found to correlate with lung function decline and persistent inflammatory activity in post-COVID-19 individuals [45]. Furthermore, recent findings suggest that anti-SARS-CoV-2 spike (anti-S) antibody levels serve as a potential biomarker for predicting long-term respiratory dysfunction in COVID-19 survivors. A large prospective study identified anti-S levels as an independent predictor of persistent symptoms and reduced lung function, with higher titers correlating with prolonged radiological lung abnormalities [46]. These findings underscore the utility of anti-S monitoring in identifying individuals at risk for prolonged pulmonary sequelae and facilitating early intervention strategies.

### 4.3. Fatigue and Chronic Inflammation

One of the most prevalent and debilitating symptoms of long COVID is chronic fatigue, which shares similarities with myalgic encephalomyelitis/chronic fatigue syndrome (ME/CFS). The biomarker soluble urokinase plasminogen activator receptor (suPAR) has been associated with sustained immune activation and endothelial dysfunction in patients with long COVID-related fatigue [47]. Increased levels of suPAR suggest ongoing low-grade inflammation that may contribute to mitochondrial dysfunction and impaired energy metabolism, providing a potential pathophysiological explanation for persistent fatigue [48].

### 4.4. Gastrointestinal Involvement in Long COVID

Gastrointestinal symptoms such as nausea, abdominal pain, and altered bowel habits have been frequently reported in long COVID. Intestinal fatty acid-binding protein (I-FABP) and zonulin have been studied as biomarkers of gut barrier integrity. Elevated I-FABP levels reflect enterocyte damage, which has been detected in COVID-19 patients with prolonged gastrointestinal symptoms [49]. Zonulin, a regulator of tight junction permeability, has been linked to increased gut permeability and systemic inflammation, possibly contributing to ongoing immune activation in long COVID patients [50].

These findings suggest that persistent inflammation and immune dysregulation are key contributors to long COVID, with distinct biomarkers associated with ongoing tissue damage in different organ systems. Future studies should aim to validate these biomarkers for clinical application, aiding in the early identification and management of post-COVID-19 complications.

## 5. Multisystem Inflammatory Syndrome in Children (MIS-C)

Multisystem Inflammatory Syndrome in Children (MIS-C) emerges as a significant post-infectious complication associated with SARS-CoV-2, characteristically presenting two to four weeks following acute COVID-19. It manifests with a constellation of symptoms including persistent fever, gastrointestinal distress, myocardial dysfunction, mucocutaneous lesions, and profound inflammatory responses [51]. Predominantly affecting children and adolescents, MIS-C has prompted concerns due to its similarity to Kawasaki disease and toxic shock syndrome, but with distinct epidemiological and clinical profiles. The exact incidence of MIS-C remains under investigation; however, it is recognized globally with variations in prevalence suggesting geographic and perhaps genetic predispositions [52,53].

The pathophysiology of MIS-C is rooted in a hyperinflammatory response, presumably triggered by an aberrant immune reaction to SARS-CoV-2. This hyperinflammation is characterized by a significant elevation of multiple inflammatory cytokines, akin to a cytokine storm, which is a hallmark of severe COVID-19 in adults but presents uniquely in children [54]. Key biomarkers of inflammation such as C-reactive protein (CRP), erythrocyte sedimentation rate (ESR), and procalcitonin are consistently elevated in MIS-C cases and serve as primary indicators of the syndrome’s severity and the extent of immune activation [55]. Additionally, elevated levels of ferritin and interleukin-6 (IL-6) further corroborate the presence of severe inflammation and are predictive of worse outcomes, underlining their role in the clinical management of the condition [51,56].

Cardiac involvement in MIS-C is particularly concerning, evidenced by elevated troponin levels and B-type natriuretic peptide (BNP), which indicate myocardial strain or injury. These cardiac biomarkers are critical for diagnosing and assessing the severity of cardiac dysfunction, which can range from mild myocarditis to severe cardiogenic shock [57]. The use of these biomarkers, combined with imaging modalities such as echocardiography, helps in guiding therapeutic decisions, particularly the administration of IV immunoglobulins (IVIGs) and steroids, which are the mainstay of MIS-C treatment [58]. Given the potential for rapid deterioration, timely intervention based on biomarker levels is crucial. Further research into the molecular mechanisms driving this severe inflammatory response may yield novel therapeutic targets that could enhance treatment strategies, potentially reducing the morbidity associated with MIS-C [59].

The identification and understanding of biomarkers in MIS-C not only facilitate immediate clinical management but also guide long-term care strategies. Immediate treatment, predominantly with IV immunoglobulins (IVIGs) and corticosteroids, has been shown to mitigate the severity of MIS-C and improve outcomes significantly [60]. As such, timely and accurate biomarker assessment is critical for initiating these therapies before the onset of irreversible damage, particularly cardiac complications [61]. Looking forward, the long-term implications of MIS-C necessitate the ongoing surveillance of recovered patients, focusing on cardiovascular health and overall physical recovery. Future research should aim to establish a standardized protocol for the long-term monitoring of children affected by MIS-C, including regular follow-ups and cardiovascular assessments to detect late sequelae early [62].

Moreover, there remains a pressing need for research into the specific pathogenic mechanisms of MIS-C, which could unveil new therapeutic targets and preventive strategies. Understanding the genetic and immunological underpinnings that predispose certain children to develop MIS-C following SARS-CoV-2 infection could lead to personalized medicine approaches, potentially offering prophylactic treatments to high-risk individuals [63]. Additionally, as new variants of the virus emerge, studying their impact on the incidence and severity of MIS-C will be crucial for adapting treatment protocols and public health policies accordingly [64].

## 6. Clinical Implications

### 6.1. Diagnostic and Prognostic Utility of Biomarkers

The utility of biomarkers in diagnosing and prognosticating COVID-19 has proven invaluable, especially in stratifying patients based on severity and potential complications. Biomarkers such as CRP, IL-6, and D-dimer have not only facilitated early detection of severe manifestations but also helped in predicting outcomes like ICU admission or the need for mechanical ventilation [65]. Their integration into clinical protocols has enabled more tailored and effective management strategies, improving patient outcomes and optimizing resource allocation in overwhelmed healthcare settings [66].

Beyond acute care, biomarkers play a crucial role in monitoring long-term recovery and identifying complications early in the post-acute phase of COVID-19. Persistent elevation of certain markers can indicate ongoing inflammation or the onset of complications such as fibrosis or cardiovascular dysfunction. Regular monitoring helps in timely intervention, potentially preventing the progression of these conditions. As such, biomarkers are integral to developing long-term care plans for COVID-19 survivors, especially those with persistent or late-emerging symptoms [67,68].

### 6.2. Therapeutic Implications and Personalized Medicine

The identification of biomarkers in long COVID has significant implications for personalized medicine, particularly in guiding treatment decisions and monitoring responses to therapy. Inflammatory markers such as IL-6, D-dimer, and CRP have been shown to correlate with disease severity and prolonged immune activation, making them valuable indicators for selecting appropriate therapeutic interventions.

IL-6, a key driver of hyperinflammation, has been targeted with IL-6 inhibitors such as tocilizumab and sarilumab, which have demonstrated efficacy in acute COVID-19 but may also have a role in managing persistent post-COVID-19 inflammatory syndromes [69,70]. Elevated D-dimer levels, indicative of ongoing coagulopathy and endothelial dysfunction, have been used to stratify patients at risk for post-COVID-19 thrombotic events, guiding decisions on extended anticoagulation therapy [71]. Similarly, CRP, a widely recognized marker of systemic inflammation, is useful in identifying patients with ongoing inflammatory responses who may benefit from corticosteroids or other immunomodulatory therapies [17,72].

Beyond inflammation, emerging biomarker-driven treatment approaches are being investigated to address specific complications of long COVID. For example, suPAR has been linked to persistent fatigue and endothelial dysfunction, prompting the exploration of therapies targeting vascular inflammation [47]. KL-6 and SP-D, both markers of alveolar epithelial damage, have been considered in assessing patients at risk of developing post-COVID-19 pulmonary fibrosis, aiding in decisions regarding antifibrotic therapy and lung rehabilitation programs [43,44]. In neurological sequelae, biomarkers such as NfL and GFAP may help clinicians monitor neuroinflammatory processes and tailor neuroprotective interventions [40].

The integration of biomarker profiling into clinical practice has the potential to personalize treatment strategies by identifying patients who are most likely to benefit from specific therapies, reducing unnecessary treatments, and improving overall patient outcomes. Moving forward, standardization of biomarker-based treatment guidelines will be essential for optimizing long COVID management and ensuring effective, individualized care.

Implementing biomarker-based strategies across diverse healthcare settings involves significant hurdles, especially in resource-limited environments. The variability in access to advanced laboratory services can limit the widespread application of novel biomarkers in routine clinical practice [73]. Moreover, the cost associated with high-throughput biomarker assays may not be feasible for all healthcare systems. Addressing these disparities through policy changes, subsidy programs, and global health initiatives is crucial for ensuring equitable healthcare delivery and maximizing the benefits of biomarker research [74].

### 6.3. Future Potential of Biomarkers in Clinical Practice

The future of biomarker research in long COVID extends beyond traditional serum-based inflammatory markers to include multisystem approaches that capture the complexity of post-viral sequelae. While conventional biomarkers such as troponin and the neutrophil-to-lymphocyte ratio (NLR) have been extensively studied in acute COVID-19, they do not represent novel indicators of long COVID-specific complications. Instead, future efforts should prioritize biomarker discovery across multiple biological systems, including respiratory, gastrointestinal, neurological, and microbiome-based assessments, to develop more holistic diagnostic and prognostic tools.

Recent studies suggest that gut microbiota composition may play a role in post-COVID-19 inflammatory regulation, with alterations in microbial diversity correlating with prolonged symptoms and immune dysfunction [75,76]. The use of microbiome-derived markers, such as short-chain fatty acids, gut permeability indicators, and microbial metabolomics profiling, could provide novel insights into chronic inflammation and systemic dysregulation in long COVID. Future research should explore the integration of microbiome-derived biomarkers with traditional serum markers to enhance risk stratification and therapeutic targeting.

Advancements in non-invasive biomarker detection, such as particle analysis in breath condensate or sputum, offer promising avenues for assessing long-term respiratory outcomes in COVID-19 survivors [77]. This method could complement traditional pulmonary biomarkers such as KL-6 and SP-D, providing a more accessible and real-time assessment of lung inflammation and recovery.

Future studies should aim to integrate multi-omics approaches, including genomics, proteomics, metabolomics, and transcriptomics, to construct a comprehensive biomarker landscape for long COVID. This system-based methodology could facilitate the identification of patient subgroups with distinct inflammatory and recovery trajectories, enabling more personalized therapeutic strategies. Additionally, artificial intelligence-driven pattern recognition in biomarker datasets could improve predictive modeling for long-term complications, optimizing clinical decision-making and resource allocation.

By expanding biomarker research into microbiome analysis, breath-based diagnostics, and multi-omics profiling, future studies can enhance our understanding of post-COVID-19 inflammatory dynamics and develop more targeted and effective interventions for long COVID management.

## 7. Future Directions

### 7.1. Research Gaps in Understanding Post-COVID-19 Biomarkers

Despite significant advances, substantial research gaps remain in our understanding of biomarkers related to post-COVID-19 conditions [78,79]. Key areas requiring further exploration include the long-term trajectory of inflammatory markers after recovery and their correlation with patient-reported outcomes. Additionally, the mechanisms through which these markers influence chronic symptoms and the potential for reversibility need comprehensive investigation. Addressing these gaps will require longitudinal studies involving diverse patient cohorts to capture the full spectrum of post-COVID-19 sequelae and their biomarker profiles [80].

Future studies should focus on validating the utility of novel biomarkers in clinical practice and their predictive power for long-term complications. Prospective cohort studies could evaluate the effectiveness of biomarker-based screening in preventing severe post-COVID-19 outcomes [81]. Moreover, intervention trials using biomarker-guided therapeutic strategies could elucidate their role in improving patient outcomes, particularly in those with persistent inflammatory states [82].

Emerging technologies, such as artificial intelligence (AI) and machine learning, are set to transform biomarker research by enhancing the analysis and interpretation of large datasets. AI-driven models could predict disease progression and outcomes based on biomarker dynamics, offering personalized treatment plans [83]. Additionally, advancements in biosensor technology may soon allow real-time monitoring of biomarkers, providing immediate feedback on patient status and treatment efficacy. These technologies not only promise to refine the detection and monitoring of biomarkers but also enable a more dynamic approach to managing post-COVID-19 conditions [84].

### 7.2. Integration of Biomarkers into Public Health Strategies

As the understanding of post-COVID-19 biomarkers advances, their integration into public health strategies becomes paramount. This integration could facilitate early identification of at-risk individuals, enabling preemptive interventions that may mitigate the development of severe post-COVID-19 complications [85]. Public health initiatives could leverage biomarker data to implement targeted health campaigns, enhance surveillance systems, and tailor community health interventions to reduce the burden of long COVID on health systems. Collaborative efforts involving epidemiologists, clinicians, and policy-makers will be essential to ensure that biomarker insights translate into actionable public health strategies [86].

With the expanding role of biomarkers in managing post-COVID-19 conditions, ethical considerations must be addressed, particularly in terms of consent, privacy, and the potential for health disparities. Ensuring that biomarker research and its clinical applications are conducted with strict adherence to ethical standards is crucial, especially when considering the implications for insurance, employment, and societal inclusion [87]. Furthermore, equity in access to biomarker-based diagnostics and treatments must be prioritized to prevent exacerbating existing health disparities within and between populations [88].

## 8. Conclusions

### 8.1. Summary of Key Insights

This review systematically explores the evolving landscape of biomarkers in the context of COVID-19, particularly focusing on their role in identifying and managing the disease and its complications, such as MIS-C. Unique to our approach is the comprehensive coverage of both established and novel biomarkers, such as suPAR and NETs, distinguishing our work from prior studies which may have focused more narrowly on specific types of biomarkers. Our analysis not only synthesizes the current knowledge but also highlights critical gaps where further research is necessary, particularly in understanding the long-term effects of COVID-19 and its biomarkers on patient health outcomes. Key findings from this review emphasize the predictive value of biomarkers like CRP, IL-6, and D-dimer in diagnosing severe cases and potential complications early, enabling timely and targeted therapeutic interventions. Looking forward, we envision a shift towards more personalized medicine approaches in managing COVID-19, underpinned by biomarker research. Future studies should focus on validating novel biomarkers and exploring their role in the long-term health management of recovered COVID-19 patients, ultimately contributing to more robust and adaptive public health strategies. Table 1 below aims to provide a comprehensive overview of the central themes addressed in our study, facilitating a clearer understanding of the context and supporting literature.

Furthermore, this review highlights the crucial role of both established and emerging biomarkers in diagnosing, monitoring, and managing post-COVID-19 conditions. Markers of inflammation and tissue damage, such as CRP, IL-6, and novel biomarkers like suPAR and NETs, have provided significant insights into the pathophysiology of COVID-19 [89]. These biomarkers have not only enhanced our understanding of the disease’s impact across various organ systems but also offered new avenues for targeted therapeutic interventions, which are vital for managing the diverse and prolonged effects of the virus [90]. A summary of these biomarkers related to inflammation, immune response, and organ damage, reflecting their critical roles in diagnosing and managing COVID-19 and its severe complications such as MIS-C, is presented in Table 2 below.

### 8.2. Clinical and Research Implications

The integration of biomarker research into clinical practice represents a paradigm shift towards more personalized medicine in the management of post-COVID-19 conditions. The continued exploration and validation of these biomarkers are imperative for refining treatment protocols and improving prognostic accuracy. Furthermore, the development of biomarker-driven models can significantly impact public health strategies by facilitating early detection and intervention, ultimately improving outcomes for individuals affected by long COVID [91,92].

### 8.3. Future Outlook and Recommendations

As the global scientific community advances in its understanding of COVID-19, the role of biomarkers in shaping future therapeutic and public health responses will undoubtedly expand [93,94]. It is recommended for future research to focus on longitudinal studies to track biomarker variations over time and explore the genetic, environmental, and clinical factors that influence these markers. Such efforts will ensure that the next steps in biomarker research not only address the current challenges but also anticipate future public health needs [95,96].

## Figures and Tables

**Table 1:** Summary of key topics and corresponding references.

Topic	Key References
Initial Immune Defense Against SARS-CoV-2	[13,14,15,16]
Dysregulation and Progression to Severe Disease	[17,18]
Chronic Inflammation and Its Consequences	[19,20,21,22,23,24]
Established Inflammatory Markers in Post-COVID-19	[25,26]
Novel Biomarkers of Inflammation	[27,28,29,30]
Systemic Inflammatory Markers and Organ-Specific Damage	[31,32,33,34]
Advances in Biomarker Detection Technologies	[35,36]
Tissue Damage and Long-Term Effects	[39,40,41,42,43,44,45,46,47,48,49,50]
Multisystem Inflammatory Syndrome in Children (MIS-C)	[51,52,53,54,55,56,57,58,59,60,61,62,63,64]
Diagnostic and Prognostic Utility of Biomarkers	[65,66,67,68]
Therapeutic Implications and Personalized Medicine	[69,70,71,72,73,74]
Future Potential of Biomarkers in Clinical Practice	[75,76,77,78]
Research Gaps in Understanding Post-COVID-19 Biomarkers	[79,80,81,82,83,84]
Integration of Biomarkers into Public Health Strategies	[85,86,87,88]

**Table 2:** Summary of known and emerging biomarkers in post-acute COVID-19 sequelae and their clinical relevance.

Biomarker	Relevance	Prognostic/Predictive Utility and Therapeutic Implications
C-reactive protein (CRP)	High levels indicate systemic inflammation	Correlates with disease severity and risk of post-COVID-19 complications. Used to guide anti-inflammatory therapy selection [63,65,66].
Interleukin-6 (IL-6)	Cytokine involved in inflammation	Elevated levels predict progression to severe COVID-19 and long COVID persistence. Targeted by IL-6 inhibitors (e.g., tocilizumab) in severe cases [55,65,66].
D-dimer	Indicates blood clot formation	Strong predictor of thrombotic complications and mortality in COVID-19. Guides anticoagulation therapy decisions [65,66].
Cardiac troponins	Markers of myocardial injury	Elevated levels indicate increased risk of cardiac sequelae post-COVID. Used for risk stratification in post-COVID-19 cardiac monitoring [42,57,61].
Ferritin	Marker of immune system activation	Correlates with hyperinflammatory states and severe outcomes. May guide immunosuppressive therapy in hyperinflammation syndromes [51,56,57].
B-type natriuretic peptide (BNP)	Marker of cardiac strain	Elevated in cases of cardiac involvement in long COVID. Used in managing post-COVID-19 cardiac dysfunction [57,59,62].
Neutrophil-to-lymphocyte ratio	Indicator of systemic inflammation	Helps assess disease severity and predict long-term immune dysregulation. Monitored as general inflammatory marker [63,78].
Procalcitonin	Marker used to evaluate bacterial infection severity	Sometimes elevated in severe COVID-19, can be used to distinguish bacterial co-infections, and supports decision-making for antibiotic therapy [2,55,64].
Erythrocyte sedimentation rate (ESR)	Indicator of inflammation level	Elevated levels indicate persistent inflammation in long COVID patients. Supports diagnosis of post-viral inflammatory syndromes [26,55,63].
Interleukin-10 (IL-10)	Anti-inflammatory cytokine	Elevated in severe COVID-19 cases; involved in immune response regulation. Potential therapeutic target in hyperinflammatory states [32,54,59].
Interferon-gamma (IFN-γ)	Immune response modulator	Elevated in severe COVID-19; indicates immune activation. May inform immunomodulatory therapies [14,61].
Interleukin-1β (IL-1β)	Pro-inflammatory cytokine	Associated with inflammatory response and cytokine storms. Targeted by IL-1 inhibitors (e.g., anakinra) in severe COVID-19 [14,18,57].
Soluble urokinase plasminogen activator receptor (suPAR)	Indicator of immune activation and potential severity	Predicts chronic inflammation and long COVID-related fatigue. Potential target for immune modulation therapies [28,54,60].
Neutrophil extracellular traps (NETs)	Components of the immune defense that trap pathogens	Elevated NETs linked to thrombosis and lung injury in COVID-19. Investigated as therapeutic target in thromboinflammation [33,34,62,78].
Krebs von den Lungen-6 (KL-6)	Marker of lung epithelial injury	Associated with post-COVID-19 pulmonary fibrosis and reduced lung function. May guide antifibrotic therapy and lung rehabilitation [43,44].
Neurofilament light chain (NfL)	Biomarker of neuronal injury	Elevated in long COVID patients with cognitive impairment. Supports monitoring of neuroinflammation and neuroprotection strategies [40,41].
Glial fibrillary acidic protein (GFAP)	Marker of astrocytic injury	Linked to persistent neurological symptoms in post-COVID-19 patients. Potential marker for long-term neuroprotection strategies [42,48].
Intestinal fatty acid-binding protein (I-FABP)	Marker of intestinal epithelial damage	Associated with gut barrier dysfunction and chronic inflammation. May guide gut-targeted therapeutic approaches [49,50].
Zonulin	Regulator of tight junction permeability	Increased levels linked to gut permeability and systemic inflammation. May serve as marker for gut-targeted post-COVID-19 therapies [49,50].
Anti-SARS-CoV-2 spike (anti-S) antibodies	Biomarker for immune response and lung recovery	Predicts prolonged respiratory dysfunction and lung inflammation. May be used to assess long-term risk for pulmonary complications [1,43,46].
